# Who Invented Vaccination?

**Published:** 1894-03-03

**Authors:** 


### Editor's Letter-Box.

### WHO INVENTED VACCINATION?

Mr. J. Collinson, of Durham, writes: In the *Evesham Journal and Four Shires Advertiser* of February 24th, 1894, publicity is given to a brief note which, I understand, appeared in a recent number of The Hospital, to the effect that at a meeting of the London Epidemiological Society Brigade-Surgeon R. Pringle, M.D., called attention "to a passage in an ancient Hindu work" as proof that vaccination was generally known—and was actually practised in India—centuries before Jenner was born. Now there are many frauds connected with the subject of vaccination—clever frauds, too. That the Hindu work, falsely (?) described as ancient, is fabulous and fraudulent, is well known to all students of vaccination history; so very well known, indeed, that it is quite unique to discover that Dr. Pringle, whose experience in matters pertaining to vaccination and its literature is very extensive—at least, that is his reputation —is in ignorance of it; and in support, and also by way of illustrating my assertion, I venture to refer you to the famous "Life of Jenner," by Dr. Baron, a warm partisan of vaccination, published in 1827, vol. I., pp. 557-559, and also to Mr. William Tebb's recently-published work on "The Recrudescence of Leprosy and its Causation," appendix pp. 354-353. Who invented vaccination? The question remains unanswered. But of this I am sure, it was not invented by Jenner, and he is no more "the great luminary" that Dr. Drysdale and others would have him appear than that anarchy will bring about the millennium. Jenner, of course, was anticipated by Benjamin Jesty, of Yetminster, who inoculated his wife and sons with cow-pox in 1774; nor was Jenner insensible to the priority of the Dorsetshire dairy-farmer's claim, but he evaded it under the plea that there was cow-pox *and* cow-pox, and that he had discovered and defined the right sort. For further particulars of Jesty and his cow-pox inoculations I must refer you to Mr. William White's "Story of a Great Delusion," pp. 94, 204, 245, and 206; and to Dr. Charles Creighton's monograph on "Jenner and Vaccination," pp. 23, 25, and 194.

